# Effectiveness of extracorporeal shock-wave therapy for frozen shoulder: A protocol for a systematic review of randomized controlled trial

**DOI:** 10.1097/MD.0000000000014506

**Published:** 2019-02-15

**Authors:** Dong-zi Cao, Cun-liang Wang, Zhong Qing, Lie-dong Liu

**Affiliations:** a Department of Orthopedics, Yangling Demonstration Zone Hospital, Yangling; b Department of Joint, Honghui Hospital, Xi’an Jiaotong University, Xi’an; c First Ward of Orthopedics Department, The First Hospital of Yulin, Yulin, China.

**Keywords:** effectiveness, extracorporeal shock-wave therapy, frozen shoulder, randomized controlled trial, safety, systematic review

## Abstract

**Background:**

This systematic review aims to explore the effectiveness and safety of extracorporeal shock-wave therapy (ESWT) for patients with frozen shoulder.

**Methods:**

The sources of Cochrane Central Register of Controlled Trials, EMBASE, MEDLINE, Cumulative Index to Nursing and Allied Health Literature, Web of Science, Allied and Complementary Medicine Database, Chinese Biomedical Literature Database, and Websites of Clinical Trials Registry will be searched. All databases and other sources will be searched from inception to the date of the search will be run. Only randomized controlled trials of ESWT for frozen shoulder will be considered for inclusion in this systematic review. Two authors independently screen the studies, extract the data, and evaluate the methodology quality for included trials. If sufficient trials will be included with fair heterogeneity, the data will be pooled, and the meta-analysis will be performed by using RevMan 5.3 software.

**Results:**

This systematic review will assess the effectiveness and safety of ESWT for frozen shoulder. The primary outcome includes pain intensity. The secondary outcomes consist of shoulder function, quality of life, and also the adverse events.

**Conclusion:**

Its findings may provide latest evidence of ESWT for the treatment of frozen shoulder.

**Ethics and dissemination:**

No research ethics approval is required in this study, because it is a systematic review and will not use individual data. The results of this study are expected to publish at peer-reviewed journals.

## 1 Introduction

Frozen shoulder, also called adhesive capsulitis of shoulder, is a common shoulder condition.^[1–3]^ Patients with this condition often complain to suffer from progressive loss of shoulder motion with painful restriction of both active and passive ranges of motion.^[4–7]^ Previous studies have reported that about 2% to 5% general population can experience this disorder.^[8–10]^ The other studies reported that more than 50% of patients with frozen shoulder still had persistent symptoms, even after several year treatments.^[11,12]^ Thus, patients with such disorder often suffer from poor quality of life.^[13,14]^

Extracorporeal shock-wave therapy (ESWT) has reported to treat a variety of pain conditions effectively and safely,^[15–21]^ including myofascial pain syndrome, knee pain, chronic pelvic pain syndrome, chronic rotator cuff tendonitis, sacroiliac joint pain, and frozen shoulder, especially the frozen shoulder.^[15–21]^ However, the effectiveness and safety of ESWT for the treatment of frozen shoulder is still inconclusive. In addition, no systematic review has addressed this issue. Thus, the protocol of this systematic review will assess the effectiveness and safety of ESWT for patients with frozen shoulder.

## 2 Methods

### 2.1 Study registration

This protocol of systematic review has been registered with PROSPERO (CRD42019120039). It has reported according to the Preferred Reporting Items for Systematic Reviews and Meta-Analysis Protocol statement guidelines.^[22]^

### 2.2 Eligibility criteria

#### 2.2.1 Study types

This protocol of systematic review will only include randomized controlled trials (RCTs) of ESWT for frozen shoulder. All other studies except the RCTs will not be included, such as animal experiments, reviews, comments, letters, expert options, case reports, case series, cross-over studies, non-RCTs, and quasi-RCTs.

#### 2.2.2 Intervention types

The treatment intervention includes ESWT only. The studies will be excluded if ESWT combined with other therapies. The control therapy can be any kinds of treatments, except the ESWT.

#### 2.2.3 Patient types

Patients with frozen shoulder, regardless of the race, sex, and age, will be included in this study.

#### 2.2.4 Outcome measurement types

Primary outcome is shoulder pain intensity. It can be measured by any kinds of pain scales. The secondary outcomes included the shoulder function and quality of life, which all can be assessed by any associated scales. Additionally, adverse events will also be examined.

### 2.3 Search strategy

We will be searching the following databases for this review without any language restrictions: Cochrane Central Register of Controlled Trials (CENTRAL), EMBASE, MEDLINE, Cumulative Index to Nursing and Allied Health Literature, Web of Science, Allied and Complementary Medicine Database, and Chinese Biomedical Literature Database. All databases and other sources will be retrieved from their inceptions up to the date of the search ran. The details of sample search strategy of CENTRAL are presented in Table 1. Similar search strategy will be used to other electronic databases. Additionally, Google scholar, clinical registration website, and reference lists of included studies will also be searched.

**Table 1:**
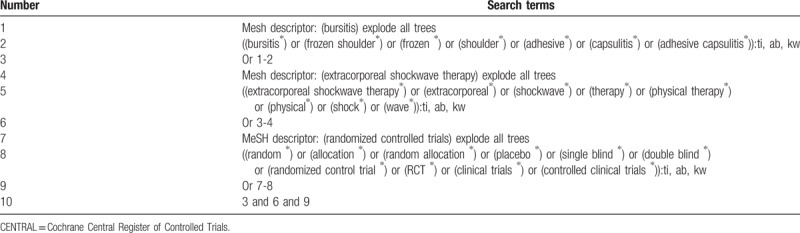
Search strategy applied in CENTRAL database.

### 2.4 Study selection process

**Figure 1:**
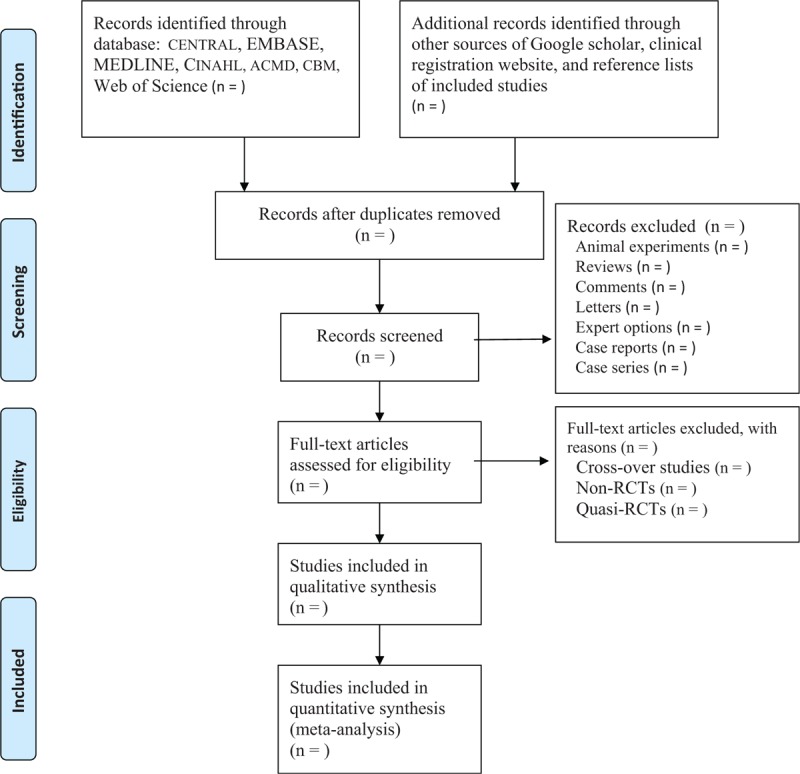
Process of study selection.

Two authors will independently select the potential literature by reading the titles and abstracts first. Then, full papers will be read if they meet all the initial criteria. If there are differences between the 2 authors, then a third author will be invited to solve them by discussion. The process of study selection is presented in Fig. 1.

### 2.5 Data collection and management process

Two independent authors will extract all the data in accordance with the previous defined data exaction form. The data of the following information will be extracted: basic information (such as authors, year of publication, region, age, and sex); study design (such as inclusion and exclusion criteria, sample size, randomization, allocation, blinding, and reporting information); intervention details in both experimental and control groups (type, session, frequency, intensity, dosage, and duration of the interventions); and outcomes (such as primary, secondary, and any other outcomes, and also the incomplete outcomes reporting). If there are disagreements between the 2 authors, a third author will be involved to resolve them by through consultation. All the extracted data will be entered into excel, and the outcome data will be pooled by using RevMan software 5.3.

### 2.6 Risk of bias evaluation

Two authors will evaluate the methodology quality of eligible studies by using Cochrane Risk of Bias Tool through 7 different domains. If there are divergences between the 2 authors, then the issues will be resolved by a third author through discussion.

### 2.7 Treatment effect measurement

In this systematic review, mean difference with 95% confidence intervals (CIs) will be applied for continuous outcome data. The risk ratio with 95% CIs will be used for dichotomous outcome data.

### 2.8 Missing data deal with

If the essential data are missing from the included studies, the original authors will be contacted for these data. If no reply is received, then the available data of included studies will be analyzed, and it will be reported in the “Discussion” section.

### 2.9 Heterogeneity evaluation and data pooled

In this systematic review, the *I*^2^ and chi-square tests will be used for the heterogeneity detection. The data will be pooled by using fixed-effect model, if *I*^2^ ≤50% is detected. On the contrary, data will be pooled by using random-effect model, if *I*^2^ > 50% is detected. In such case, subgroup analysis will be performed to identify any potential factors that may cause the heterogeneity. If the heterogeneity is still considerable after the subgroup analysis, the data will not be pooled, but a narrative summary will be presented instead. The meta-analysis will be conducted if the heterogeneity is reasonable, normally with *I*^2^ ≤50%, or potential factors that may result in heterogeneity after subgroup analysis with *I*^2^ > 50%.

### 2.10 Subgroup analysis

Subgroup analysis will be conducted to identity any potentials reasons that may cause heterogeneity if the value of *I*^2^ is more than 50%. It will be performed based on the different types of experimental and control interventions, or the outcome measurement scales.

### 2.11 Sensitivity analysis

If sufficient studies will be included, and data will be pooled, then sensitivity analysis will be considered for detecting the robustness of pooled outcome results, methodological quality, and also the potential effects of missing data.

### 2.12 Publication bias

In this systematic review, funnel plot will be conducted to detect any possible publication biases if more than 10 RCTs are included,^[23]^ and Egg regression test will be performed to check the funnel plot asymmetry.^[24]^

Patient and public involvement: Neither patient nor public will be involved in this work.

## 3 Discussion

Researchers hypothesize that ESWT plays a critical role in the treatment of patients with frozen shoulder. However, until present, the literature on ESWT focused on frozen shoulder largely has been conceptual. Given emerging literature on ESWT for frozen shoulders, we aim to conduct a systematic research synthesis to inform the effectiveness and safety of ESWT for frozen shoulder. We are expected to establish the current knowledge base regarding the effectiveness and safety of ESWT for the treatment of frozen shoulder. The results of the present systematic review will be disseminated to a variety of stakeholders interested in ESWT therapy to inform both the researchers for the further studies and clinical practice focused on the public health approach to education.

## Author contributions

**Conceptualization:** Dong-zi Cao, Cun-liang Wang, Lie-dong Liu.

**Data curation:** Dong-zi Cao, Zhong Qing, Lie-dong Liu.

**Formal analysis:** Dong-zi Cao, Zhong Qing.

**Investigation:** Dong-zi Cao.

**Methodology:** Dong-zi Cao, Cun-liang Wang, Zhong Qing, Lie-dong Liu.

**Project administration:** Lie-dong Liu.

**Resources:** Dong-zi Cao, Cun-liang Wang, Zhong Qing, Lie-dong Liu.

**Software:** Dong-zi Cao, Cun-liang Wang, Zhong Qing.

**Supervision:** Dong-zi Cao.

**Validation:** Dong-zi Cao, Cun-liang Wang, Zhong Qing, Lie-dong Liu.

**Visualization:** Zhong Qing, Lie-dong Liu.

**Writing – original draft:** Dong-zi Cao, Cun-liang Wang, Zhong Qing, Lie-dong Liu.

**Writing – review & editing:** Dong-zi Cao, Cun-liang Wang, Zhong Qing, Lie-dong Liu.
